# Correction: Synthesis of an amantadine-based novel Schiff base and its transition metal complexes as potential ALP, α-amylase, and α-glucosidase inhibitors

**DOI:** 10.1039/d3ra90035e

**Published:** 2023-04-17

**Authors:** Aliya Ajaz, Muhammad Ashraf Shaheen, Maqsood Ahmed, Khurram Shahzad Munawar, Abu Bakar Siddique, Abdul Karim, Nazir Ahmad, Muhammad Fayyaz ur Rehman, Tahir Mehmood

**Affiliations:** a Institute of Chemistry, University of Sargodha 40100 Pakistan aliyarajput2822@gmail.com ashraf.shaheen@uos.edu.pk fayyaz9@gmail.com abubakar.siddique@uos.edu.pk abdul.karim@uos.edu.pk; b Materials Chemistry Laboratory, Institute of Chemistry, The Islamia University of Bahawalpur Baghdad-ul-Jadeed Campus 63100 Pakistan maqsood.ahmed@iub.edu.pk; c Department of Chemistry, University of Mianwali Mianwali 42200 Pakistan khurramchemist@gmail.com; d Department of Chemistry, Government College University Lahore Lahore 54000 Pakistan nazirphysical@gmail.com; e Centre for Applied Molecular Biology (CAMB), University of the Punjab Lahore-53700 Punjab Pakistan tahir.camb@pu.edu.pk

## Abstract

Correction for ‘Synthesis of an amantadine-based novel Schiff base and its transition metal complexes as potential ALP, α-amylase, and α-glucosidase inhibitors’ by Aliya Ajaz *et al.*, *RSC Adv.*, 2023, **13**, 2756–2767, https://doi.org/10.1039/D2RA07051K.

Following an institutional investigation by the University of Sargodha, the authors regret the omission of Tahir Mehmood in the original author list.

The correct authorship list and affiliations list is displayed herein. The updated acknowledgements and author contributions are shown below.

## Author contributions

Conceptualization, Aliya Ajaz and Muhammad Ashraf Shaheen; methodology, Muhammad Ashraf Shaheen, Aliya Ajaz, Abu Bakar Siddique; software, Maqsood Ahmed; data collection, structure solution, refinement, validation, Khurram Shahzad Munawar, Muhammad Fayyaz ur Rehman and Abdul Karim; formal analysis, Nazir Ahmad; investigation, Abdul Karim; resources, Muhammad Ashraf Shaheen; data curation, Aliya Ajaz; writing—original draft preparation, Aliya Ajaz; writing—review and editing, Abu Bakar Siddique; visualization, Muhammad Fayyaz ur Rehman; supervision, Muhammad Ashraf Shaheen, Tahir Mehmood; project administration, Muhammad Ashraf Shaheen. All authors have read and agreed to the published version of the manuscript.

## Acknowledgements

We are thankful to the Higher Education Commission of Pakistan (HEC) for providing financial assistance to PhD Scholar (Aliya Ajaz) to conduct a part of her research work in the United States under the International Research Support Initiative Program (IRSIP).

The Royal Society of Chemistry apologises for these errors and any consequent inconvenience to authors and readers.

## Supplementary Material

